# Sulindac plus a phospholipid is effective for polyp reduction and safer than sulindac alone in a mouse model of colorectal cancer development

**DOI:** 10.1186/s12885-020-07311-4

**Published:** 2020-09-10

**Authors:** Jennifer S. Davis, Preeti Kanikarla-Marie, Mihai Gagea, Patrick L. Yu, Dexing Fang, Manu Sebastian, Peiying Yang, Ernest Hawk, Roderick Dashwood, Lenard M. Lichtenberger, David Menter, Scott Kopetz

**Affiliations:** 1 Departments of Epidemiology, The University of Texas, MD Anderson Cancer Center, PO Box 301439, Houston, TX 77230-1439 USA; 2 Departments of Gastrointestinal Medical Oncology, University of Texas, MD Anderson Cancer Center, Houston, TX USA; 3 Departments of Veterinary Medicine and Surgery, University of Texas, MD Anderson Cancer Center, Houston, TX USA; 4 McGovern Medical School, University of Texas Health Science Center, Houston, TX USA; 5 Departments of Epigenetics & Molecular Carcinogenesis, University of Texas, MD Anderson Cancer Center, Houston, TX USA; 6 Departments of Palliative, Rehabilitation and Integrative Medicine, University of Texas, MD Anderson Cancer Center, Houston, TX USA; 7 Division of Cancer Prevention and Population Sciences, University of Texas, MD Anderson Cancer Center, Houston, TX USA; 8 Center for Epigenetics & Disease Prevention, Institute of Biosciences and Technology, Texas A&M University, Houston, TX USA

**Keywords:** Colorectal cancer, Chemoprevention, Gastrointestinal safety, Sulindac, Polyps

## Abstract

**Background:**

Non-steroidal anti-inflammatory drugs (NSAIDs) such as aspirin and sulindac are effective for colorectal cancer prevention in humans and some animal models, but concerns over gastro-intestinal (GI) ulceration and bleeding limit their potential for chemopreventive use in broader populations. Recently, the combination of aspirin with a phospholipid, packaged as PL-ASA, was shown to reduce GI toxicity in a small clinical trial. However, these studies were done for relatively short periods of time. Since prolonged, regular use is needed for chemopreventive benefit, it is important to know whether GI safety is maintained over longer use periods and whether cancer prevention efficacy is preserved when an NSAID is combined with a phospholipid.

**Methods:**

As a first step to answering these questions, we treated seven to eight-week-old, male and female C57B/6 *Apc*^*min/+*^ mice with the NSAID sulindac, with and without phosphatidylcholine (PC) for 3-weeks. At the end of the treatment period, we evaluated polyp burden, gastric toxicity, urinary prostaglandins (as a marker of sulindac target engagement), and blood chemistries.

**Results:**

Both sulindac and sulindac-PC treatments resulted in significantly reduced polyp burden, and decreased urinary prostaglandins, but sulindac-PC treatment also resulted in the reduction of gastric lesions compared to sulindac alone.

**Conclusions:**

Together these data provide pre-clinical support for combining NSAIDs with a phospholipid, such as phosphatidylcholine to reduce GI toxicity while maintaining chemopreventive efficacy.

## Background

Aspirin and non-aspirin non-steroidal anti-inflammatory drugs (NSAIDs) are increasingly recognized as effective chemoprevention agents against colorectal cancer (CRC) [1, 2]. However, the broader use of aspirin for CRC prevention is greatly limited due to the significant risk of gastro-intestinal (GI) ulceration and bleeding resulting from prolonged, regular use in humans [3]. In the cardiovascular disease (CVD) prevention realm, several strategies have emerged to reduce the risk of gastric ulceration, including enteric coatings [4] and co-administration with a proton pump inhibitor (PPI) [5]. In the case of enteric coatings, they do not always lower-GI injury [6] and may interfere with the anti-platelet effects of aspirin [7]. Although effective for GI injury prevention, the long-term safety of PPIs has recently come into question [8], limiting consumer options for GI protection from NSAID induced GI injury.

One potential mechanism for GI injury is disruption of the hydrophobic gastric surface mucosa by aspirin and non-aspirin NSAIDs, exposing the epithelium to gastric acid, leading to ulceration [9–11]. The addition of a phospholipid [10–12] to aspirin and non-aspirin NSAIDs may reduce disruption of the hydrophobic mucosa and holds promise as an emerging strategy to reduce gastric ulceration. Moreover, the combination of aspirin with the phospholipid phosphatidylcholine (PC) recently attained FDA approval following a successful clinical trial demonstrating bioequivalence to immediate-release aspirin [13]. Importantly, a separate, successful clinical trial demonstrated significantly reduced gastric ulceration in participants receiving PL-ASA (aspirin plus PC) compared to those receiving immediate-release aspirin [14]. Although these results are promising, PL-ASA has not been commercially available for a sufficient time to demonstrate long-term safety with prolonged use, as is needed for CRC prevention benefit [2]. Further, although PL-ASA has equivalent anti-pyretic, anti-inflammatory and anti-platelet properties as traditional aspirin, its chemopreventive properties are still being evaluated in vivo [15].

Sharing genetic etiology with the human Familial Adenomatous Polyposis (FAP) syndrome, the *Apc*^*min/+*^ mouse harbors a heterozygous truncating mutation in the *Apc* gene, leading to the formation of many polyps throughout the intestinal tract. Unlike humans with FAP, *Apc*^*min/+*^ mice develop most of their lesions in the small intestine, with infrequent development of colon tumors. Also, in contrast to FAP patients, *Apc*^*min/+*^ mice rarely progress to adenocarcinoma, instead becoming moribund due to intestinal polyp burden and resulting anemia. Despite these dissimilarities, this model has proven useful for testing many chemopreventive agents including non-aspirin NSAIDs, the selective cyclooxygenase-2 (COX-2) inhibitor celecoxib, curcumin, and fish oil [16–21].

While NSAIDs such as sulindac, ibuprofen and piroxicam have demonstrated consistent efficacy in this model, aspirin studies in *Apc*^*min/+*^ mice have yielded mixed results [22–27]. Based on consistent findings of chemopreventive benefit of sulindac for humans with FAP [28] and faithfulness of the mouse model to recapitulate this benefit [21], we conducted studies in *Apc*^*min/+*^ mice to test the chemopreventive efficacy and safety of sulindac pre-associated with PC. To our knowledge, this is the first report of sulindac combined with PC.

## Methods

### Animals

All procedures were reviewed and approved by MD Anderson’s Institutional Animal Care and Use Committee. *Apc*^*min/+*^ mice on the C57B/6 background were previously obtained from JAX (stock 002020) and a local breeding colony was established in a specific pathogen free environment. Mice were group housed in individually ventilated cages with a HEPA filtered air supply and blower exhaust. All cages had corn cob bedding and a Nestlet® for enrichment. To the extent possible, mice were group housed with 2–3 animals per experimental cage. In the rare instances where individual housing was necessary, due to fighting, mice were provided a paper hut in addition to the Nestlet®. Chlorinated, reverse osmosis water was provided ad libitum via a valve at the rear of the cage. Mice were provided with Purina PicoLab Rodent Diet (5053, Purina), ad libitum. At 7 to 8 weeks of age (mean = 8.0, range: 7.4–8.4), male and female mice (mean weight = 20.7 g, range: 15.9–25.4) were randomized to receive one of three controls, or one of two treatments (Table 1). The control groups included no treatment (6 mice), PBS (7 mice), and PC (volume equivalent to 30 mg/kg sulindac, 7 mice). The treatment groups included sulindac (30 mg/kg, 7 mice) and sulindac-PC (30 mg NSAID/kg, 6 mice). A sulindac dose of 30 mg/kg per day was chosen based on prior experience and approximates 150 mg/day in an adult human [29]. Sample size was chosen based on a power calculation to detect a 40% decrease in intestinal polyp count between untreated and sulindac treated mice at the end of study. With a minimum sample size of 6 per group, we had 80% power to detect a 40% decrease at a *p* value of 0.01. Polyp burden, defined as total intestinal polyp area, was also assessed. Mice were administered PBS, PC, sulindac or sulindac-PC by daily oral gavage, using a soft-tip flexible gavage needle (Instech, FTP1838) for 3 weeks. Treatment length of 3 weeks was chosen as the time needed to reduce intestinal polyp count by at least 40%, which was the basis of our power calculation. Treatments were conducted in the morning in the animal’s home cage. Daily oral gavage was chosen, as it more closely resembles the mechanism of exposure in humans. Mice randomized to no treatment were restrained daily to control for the stress of daily manual restraint. Randomization and study entrance were conducted on a rolling basis, as animals became available, aiming to balance sex and age within each treatment or control group. Sulindac (Sigma) from a single batch was either combined with PC ((Lipoid S 100) Lipoid GmbH, Germany) as previously described [30] or on its own was diluted in PBS to a working concentration of 5 mg/mL and sonicated in a sonicating water bath for 30 min at room temperature (Branson 1800). The individual structures of sulindac (CAS: 38194–50-2) and PC (CAS: 97281–47-5) are known and have been previously published [15, 31]. Fresh aliquots were prepared each day prior to administration. Mice were weighed twice weekly and monitored for overall health condition and dose levels were adjusted once per week based on weight. At the completion of the treatment course, mice were euthanized via carbon dioxide asphyxiation, followed by cervical dislocation. Blood and urine samples were collected, and necropsy was performed in all mice. During necropsy the stomach was examined and evaluated for the presence of ulcers, and the intestinal tract for presence of mucosal polyps. Briefly, the intestinal tract, from the duodenum to the rectum, was excised in-tact, flushed with PBS, expanded with freshly prepared 1.1% paraformaldehyde, 1.25%glutaraldehyde (in PBS) and fixed in this solution at 4 °C for 72 h. Mouse treatment identification was blinded at necropsy, where each mouse was assigned a 5-digit, non-sequential number. Animal ids remained blinded to all data analysts until measurements were completed.

**Table 1:** Baseline animal characteristics

	No Treatment	PBS	PC	Sulindac	Sulindac-PC
n	6	7	7	7	6
Sex n (%)					
male	3 (50)	4 (57)	4 (57)	4 (57)	2 (33)
female	3 (50)	3 (43)	3 (43)	3 (43)	4 (67)
Age, weeks (SD)	8.2 (0.2)	8.0 (0.2)	8.1 (0.2)	7.8 (0.1)	7.9 (0.2)
Weight, grams (SD)	20.4 (2.2)	20.5 (3.5)	21.7 (3.2)	21.0 (3.2)	19.9 (3.6)

### Polyp evaluation

Following 72 h of fixation, the fixative was drained from each intestinal tract and the tissue was transferred to 70% ethanol and maintained at 4 °C until analysis. For analysis, each intestinal tract was split longitudinally, spread open with mucosal surface exposed for observation and photographed in PBS on a Nikon SMZ1500 dissecting microscope by investigators blinded to the animal treatment condition. Micrographs of the entire mucosal surface were separated into manageable segments and evaluated for the presence of abnormal lesions that were clearly distinguishable from Peyer’s Patches. Lesions were marked and measured using NIS elements software (Nikon). Following completion of polyp annotation and measurement, the animal treatment conditions were un-blinded and summary statistics generated comparing total polyp number, total polyp area and polyp size per treatment condition by ANOVA followed by Tukey’s HSD post-test.

### Gastric gross and histological assessment

At necropsy, the stomach was removed from each animal and opened along the greater curvature for exposure of the gastric mucosa. The tissue was gently rinsed with PBS, examined grossly and photographed using a dissecting microscope. Then all stomachs were fixed in 10% neutral buffered formalin for 48–72 h. Multiple sections from each formalin fixed stomach were processed and embedded in paraffin blocks. Four-micron-thick sections of these tissue blocks were stained with hematoxylin and eosin (H&E) and examined microscopically by a veterinary pathologist without knowledge of animal treatment identification. The severity and extent of histopathological lesions of inflammation and hyperplasia of gastric mucosa were scored with either grade 1 (minimal lesions affecting 1–10% of tissue), grade 2 (mild lesions affecting 11–20%), grade 3 (moderate lesions affecting 21–40%) or grade 4 (marked lesions affecting 41–100% of examined tissue). Ulceration (loss of entire mucosal thickness) of the glandular gastric mucosa was recorded as either present or absent. The highest-grade inflammatory lesion (1–4) was plotted for each mouse and group differences were assessed by *Student’s* t test.

### Immunohistochemistry evaluation

After imaging completion, fixed intestinal tissues were placed in a modified Swiss roll formation, and embedded in paraffin for sectioning. Paraffin sections of the small and large intestine were stained by routine H&E protocol. Purified mouse anti β catenin antibody (# 610153) was purchased from BD Bioscience (San Jose, CA) and intestine sections were stained as per the protocol validated by the Research Histology Pathology Imaging Core of MDACC. The β catenin staining intensity of each polyp was scored at a scale of 0 to 3, 0-no staining, 1+ = weak staining, 2+ = mild staining, 3 + = strong staining. H-scores were generated for each mouse using the following formula: H = [1 × (% polyps 1+) + 2 × (% polyps 2+) + 3 × (% polyps 3+)]. Both H-scores and average percent polyps per staining intensity are shown. Histopathology evaluation was conducted without knowing the identity of the specimens with respect to treatment and group assignment. Group comparisons were conducted on the H-score data using ANOVA followed by Tukey’s HSD post-test. Graphs and statistical analyses were produced using GraphPad Prism 7.03 (GraphPad Software, Inc.).

### Hematologic evaluation

Blood samples collected at euthanasia were tested for complete blood cell counts (red blood cells, white blood cells, platelets, hemoglobin and hematocrit) with differential counts (neutrophils, eosinophils, segmented cells, monocytes and lymphocytes) and blood chemistry tests (blood urea nitrogen (BUN), creatinine, alkaline phosphatase, alanine transaminase (ALT), aspartate aminotransferase (AST), albumin, globulin, total protein and total bilirubin). Blood counts and chemistries were compared for differences across treatment groups using ANOVA followed by Tukey’s HSD post-test.

### Urine prostaglandins

Urinary prostaglandins are a frequently used measure of systemic COX activity, as they are down-stream metabolites of these enzymes [32]. Urine was collected from the euthanasia chamber and puncture of the urinary bladder at necropsy. For mice with insufficient urine collected, samples were pooled by treatment. Following collection, 100 μl urine aliquots were immediately frozen on dry ice and maintained at -80C until the time of analysis. Urinary prostaglandin profiles were measured using an Agilent 6460 triple quadrupole chromatograph/mass spectrometer as previously described [33]. Briefly, 50 μl of urine was spiked with 100 ng tetraor PGEM-d6, 2,3-dinor-PGF1α-d9, and 11-dehydrox-TXB2-d4 (internal standards for urinary metabolites of PGE_2_, PGI_2_, and TXB_2_) followed by derivatization with methanoxyamine hydrochloride solution (25 μg). Samples were then incubated at 37 °C for 30 min. The urinary metabolites were applied to Strata-X (30 mg) reverse phase extraction cartridges (Phenomenex, Milford, MA), eluted with 5% acetonitrile (ACN) in ethyl acetate, dried with a stream of nitrogen followed with reconstitution in 100 μl of 50% methanol water. To fully quantify urinary COX-2 metabolites, these metabolites were separated by reversed-phase HPLC (Agilent 1200, Santa Clara, CA) using Phenomenex Kinetex C18 column (100 mm × 2.1 mm I.D., 2.6 μm) with gradient mobile phase of 0.05% aqueous acetic acid and 0.05% acetic acid in methanol: ACN (5:95). The identification and quantification of these urinary metabolites were carried out using Agilent 6460 triple quadruple mass spectrometer by negative multiple reaction, monitoring the transition of PGEM at *m*/*z* 385 ➔ 336, PGIM set at *m*/*z 370* ➔ 232 and TXBM at m/z 370 ➔ 155. Creatinine levels were used to normalize the final outcome of the urinary COX-2 metabolites.

## Results

**Fig. 1:**
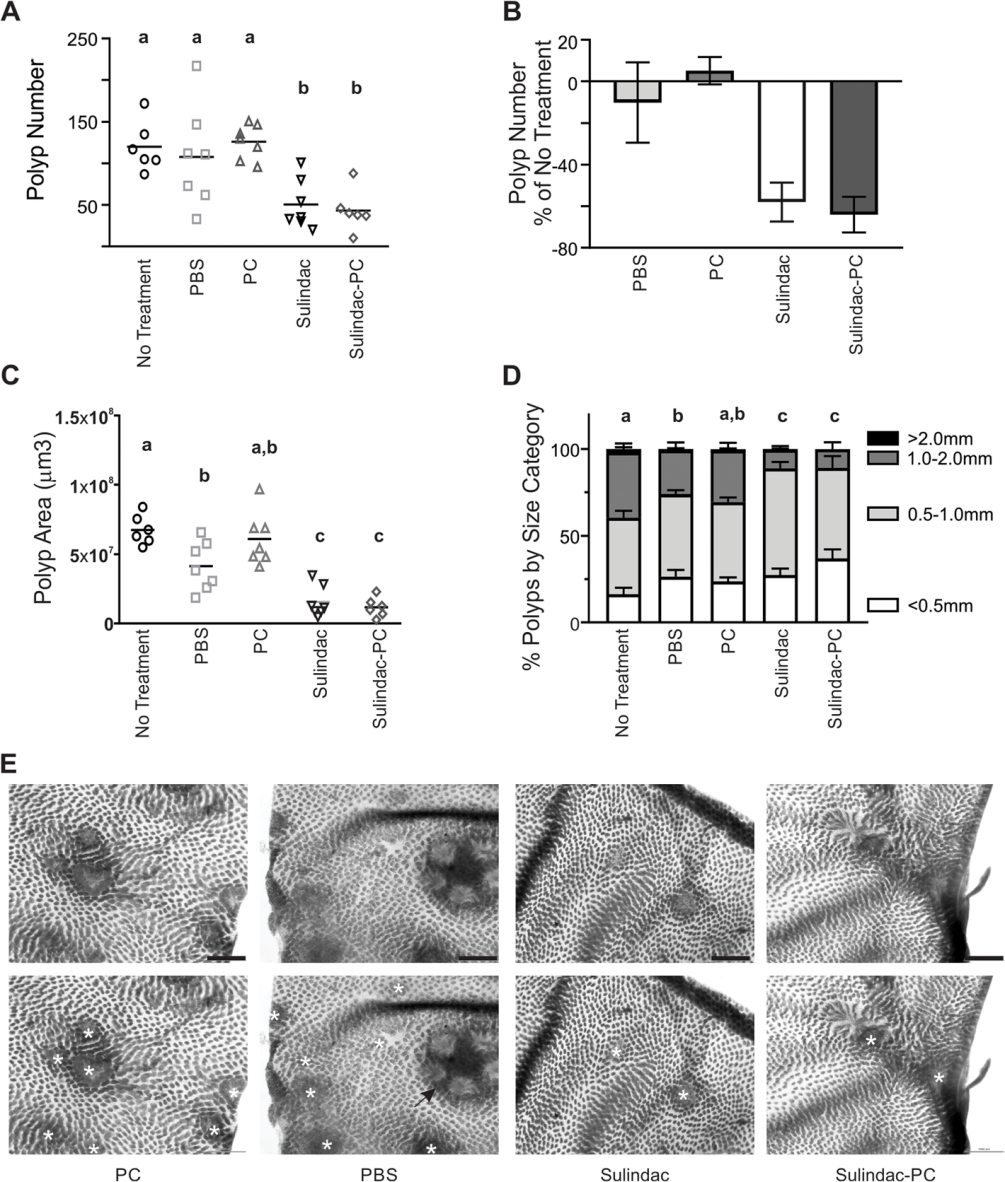
Sulindac and Sulindac-PC are effective at reducing polyp burden. **a** Total intestinal polyp number by treatment group No Treatment (**○**), PBS (**□**), PC (∆), Sulindac (), Sulindac-PC (◊), groups with different letters are significantly different from each other. Each point represents data from an individual mouse. **b** Percent reduction in polyp count compared to No Treatment group. **c** Total intestinal polyp area by treatment group **d** Percent of polyps are shown by size category and treatment group, groups with different letters have significantly different proportions of 1.0–2.0 mm polyps. Columns = average, bars = standard error of the mean, *N* = 6–7 mice per treatment group, each symbol represents one mouse **e** Representative images of polyp annotations. Tissues are shown without (top) and with (bottom) polyps annotated (*). Arrow indicates a Peyer’s patch. Scale bar = 1000 μm

We tested the relative ability of sulindac and sulindac-PC to reduce polyp count and polyp burden in *Apc*^*min/+*^ mice treated for 3 weeks starting at 7 to 8 weeks of age. Two mice randomized to the sulindac-PC arm became moribund very early in the experiment due to gavage accident and were euthanized. Gavage techniques were re-optimized to avoid any further injury. These mice were replaced in the study and their data are not included in the analyses. No other adverse events were observed. Mice treated with either sulindac or sulindac-PC had significantly reduced polyp count (Fig. 1a). Specifically, polyp count was reduced by approximately 58% with sulindac treatment and 64% with sulindac-PC treatment (Fig. 1b). Polyp burden, as measured by intestinal polyp area, was significantly reduced compared to non-treated and PC only treated animals (Fig. 1c). Additionally, the size of the remaining polyps tended to be smaller with significantly lower percentages of 1.0–2.0 mm polyps in sulindac and sulindac-PC treated mice compared to controls (Fig. 1d). Representative intestinal images are shown with and without polyp annotations (Fig. 1e).

### GI safety

**Fig. 2:**
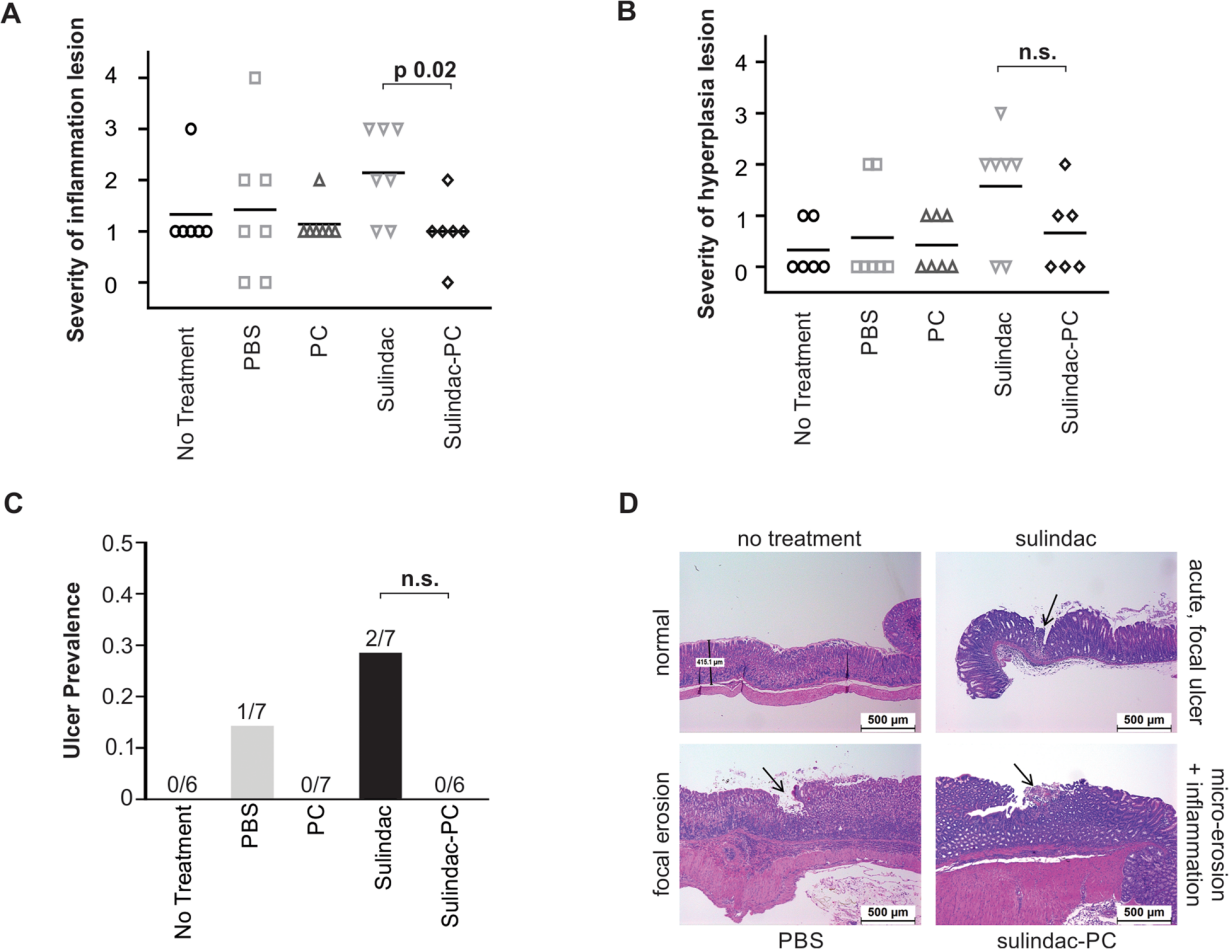
Treatment with Sulindac-PC results in significantly less gastric toxicity compared to Sulindac. **a** Gastric Inflammation by treatment. **b** Hyperplasia of the glandular epithelium by treatment. **c** Ulcer Prevalence by treatment. **d** Example images of scored lesions, arrows indicate specified lesions. Scale bar = 500 μm. No Treatment (**○**), PBS (**□**), PC (∆), Sulindac (), Sulindac-PC (◊)

Stomachs were examined histopathologically for the presence of gastric lesions, which were graded as described above and compared across treatment conditions. Histopathologic lesions observed include: acute and subacute ulceration of the glandular mucosa, and acute and subacute inflammation of the gastric glandular mucosa and submucosa. Lesions of inflammation and hyperplasia of epithelial cells of glandular mucosa indicate mucosal injury and/or healing of preexistent mucosal erosions or ulcers caused by sulindac treatment or stress. Inflammation of glandular gastric mucosa was observed in 7/7 mice from sulindac treated group and in 5/6 mice from sulindac-PC treated group. The severity of inflammation of gastric mucosa was significantly greater in the sulindac treated group (2.14 average score) in comparison with the sulindac-PC treated group (1.00 average score, Fig. 2a, *p* = 0.02). Similarly, the incidence and severity of hyperplastic changes of glandular epithelium of gastric mucosa was higher in the sulindac treated group (5/7 mice and 1.57 average score) in comparison with sulindac-PC treated group (3/6 mice and 0.67 average score), though this difference in scores was not statistically significant (*p* = 0.13) (Fig. 2b).

Histopathological examination revealed presence of ulceration of gastric mucosa in 2/7 mice treated with sulindac alone, while none of the six mice treated with sulindac-PC had lesions of ulceration, though this difference was not statistically significant (Fig. 2c). Representative photomicrographs of scored lesions are displayed by treatment condition (Fig. 2d), showing normal gastric mucosa in a mouse receiving no treatment (upper left), an acute, focal ulcer in a mouse receiving sulindac alone (upper right, arrow), a focal erosion of the gastric mucosa in a mouse receiving PBS (lower left, arrow), and a micro-erosion of the gastric mucosa with inflammation in a mouse receiving sulindac-PC (lower right, arrow).

### Biological activity of sulindac and sulindac-PC

**Fig. 3:**
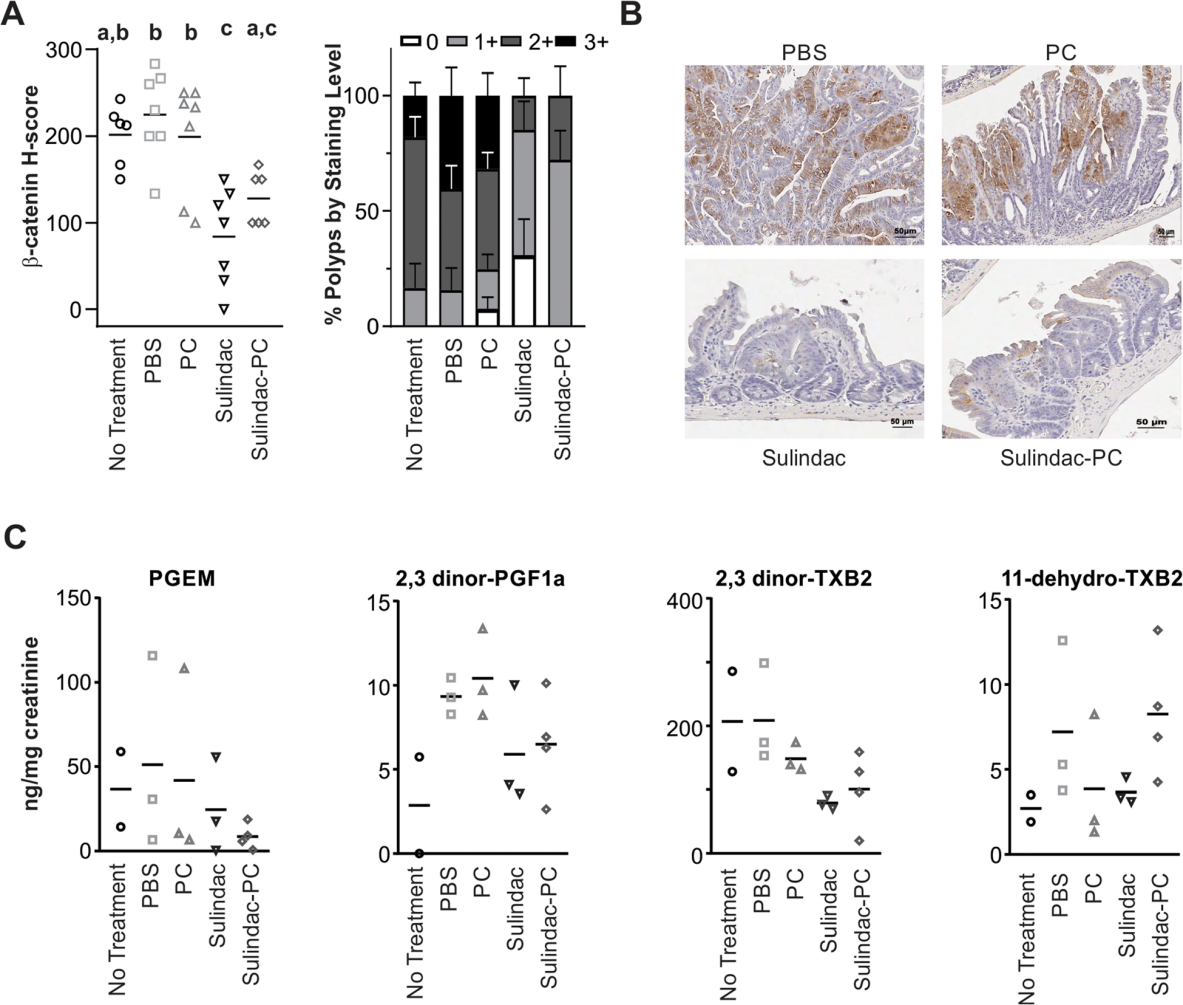
Sulindac and Sulindac-PC show biological activity. **a** Nuclear β-catenin IHC scores by treatment (left) and percent lesions at each staining level (right) columns = mean, bars = standard error. Groups with different letters are significantly different from each other **b** Representative images of β-catenin staining and localization within polyps. Scale bar = 50 μm. **c** End of study urinary prostaglandin profiles by treatment group. No Treatment (**○**), PBS (**□**), PC (∆), Sulindac (), Sulindac-PC (◊)

In addition to polyp reduction, the biological activity of sulindac and sulindac-PC was evaluated by measuring the relative intensity of nuclear β-catenin staining by immunohistochemistry (IHC), as an indicator of cellular proliferative activity (Fig. 3a-b). Of the polyps remaining in the sulindac and sulindac-PC treatment groups, there was significantly less nuclear β-catenin staining compared to controls. Sulindac, like other NSAIDs inhibits the cyclooxygenase (COX) pathway and specifically inhibition of COX-2 may be important for CRC prevention [32]. To assess systemic effects of sulindac and sulindac-PC treatment, we assessed endpoint urinary prostaglandin levels, down-stream metabolites of COX activity, across treatment groups, showing reductions in PGEM and 2,3 dinor-TXB2 in the sulindac and sulindac-PC treated animals with some variability in reductions of additional prostaglandins measured (Fig. 3c).

### Animal health

Treatment did not alter weight gain trajectory of treated mice compared to the non-treated control (data not shown). At the end of each study, blood chemistries (including liver and kidney function tests) and complete blood counts were obtained (Table 2). Differences were noted in the complete blood count between control and sulindac or sulindac-PC treated animals, including increased hematocrit and red blood cell counts and increased mean corpuscle hemoglobin concentration (Table 2).

**Table 2:** Summary hematology results

**Blood Chemistry results by treatment group**						
Blood Chemistry	No Treatment	PBS	PC	Sulindac	Sulindac-PC	*p* value
n	6	7	7	6	5	
Albumin (SD)	3.5 (0.3)	3.4 (0.3)	3.3 (0.4)	3.7 (0.2)	3.5 (0.1)	0.3
Alk Phos	109.8 (25.4)	101.9 (24.5)	92.6 (23.5)	119.0 (25.8)	88.2 (29.1)	0.3
ALT	124.7 (131.4)	350 (352.6)	237.7 (267.3)	125.7 (42.1)	95.8 (62.2)	0.3
AST	287.8 (86.4)	514.3 (500.8)	650.1 (424.0)	268.7 (99.3)	416.5 (471.8)	0.3
n	6	7	7	6	4	
BUN	23.4 (4.3)	27.1 (4.7)*	18.6 (3.4)*	24.1 (4.3)	24.3 (6.3)	**0.03**
Globulin	1.4 (0.3)	1.4 (0.2)	1.5 (0.2)	1.5 (0.2)	1.4 (0.1)	0.7
Total Protein	4.9 (0.6)	4.9 (0.4)	4.8 (0.4)	5.2 (0.3)	5.0 (0.3)	0.3
n	2	1	2	4	4	
Creatinine	0.24 (0.04)	0.20	0.24 (0.01)	0.24 (0.02)	0.22 (0.02)	0.5
n	0	1	1	0	1	
Total Bilirubin		0.2	0.3		0.2	NA
**Complete Blood Counts by treatment group**						
Blood Chemistry	No Treatment	PBS	PC	Sulindac	Sulindac-PC	*p* value
n	6	7	7	6	6	
Hemoglobin, g/dL (SD)	13.5 (2.5)	13.4 (0.8)	13.2 (1.8)	15.7 (1.2)	15.3 (1.3)	0.03
Hematocrit, %	49.6 (9.0)	**48.1**^**a**^ **(3.3)**	**47.7**^**a**^ **(5.9)**	**58.3**^**b**^ **(4.7)**	53.2 (3.9)	0.01
RBC count, x10e^6^/μL	9.6 (2.0)	9.3 (0.7)*	9.5 (1.3)	11.5 (0.7)*	10.8 (0.8)	0.01
WBC count, x10e^3^/μL	8.7 (1.6)	5.7 (2.0)	6.9 (2.7)	9.4 (3.7)	7.5 (2.4)	0.11
Platelet count, x10e^3^/μL	881 (375)	1049 (100)	1056 (323)	644 (310)	777 (394)	0.11

Values marked with * are significantly different from each other, but not any other values in that row. Values with different superscript letters are statistically different from each other. For example, columns with ‘a’ are significantly different from columns with ‘b’, or ‘c’, but not different from other columns with ‘a’. For example, hematocrit percentages for PBS and PC treated animals are significantly lower than sulindac treated animals, but are not different from each other. *ALT* Alanine transaminase, *AST* Aspartate aminotransferase, *Alk Phos* Alkaline phosphatase, *BUN* Blood urea nitrogen, *n* number of animals, *RBC* Red blood cells, *WBC* White blood cells

## Discussion

For effective chemoprevention strategies to be accepted and utilized, the benefits of such treatments must significantly outweigh the risks. Based on substantial concerns over GI toxicity and bleeding, aspirin use for CRC chemoprevention is restricted to relatively small populations. Improving the GI safety of aspirin and non-aspirin NSAIDs is an important step to making these agents safer for chemopreventive use in larger populations. Our findings of decreased stomach toxicity in sulindac-PC mice compared to sulindac alone supports the hypothesis that associating NSAIDs with phospholipids, such as phosphatidylcholine may be an important strategy to minimize the GI toxicity of prolonged use without compromising chemopreventive efficacy.

These observations clearly demonstrate that sulindac-PC treatment resulted in significantly decreased gastric inflammation and may result in decreased hyperplasia of gastric mucosa and ulceration compared with sulindac alone (Fig. 2). The differences in severity of hyperplasia were suggestive of improvements in sulindac-PC, but severity of lesions within treatment groups was heterogenous (Fig. 2b). Further, the very low prevalence of ulcers in the treatment groups did not provide adequate power to detect a significant difference between treatment groups. Several factors likely contributed to these non-significant differences. First, our dose of sulindac (30 mg/kg/day) was chosen as the dose needed to reduce polyps with 3 weeks of daily dosing, but is equivalent to approximately half of the daily dose [29] used in a primary chemoprevention trial with FAP patients, who were given 150 mg of sulindac twice daily [34]. Second, the length of treatment in our study was relatively short. Increasing sulindac dose, length of treatment, or both may increase the prevalence of gastric injury observed. Despite these limitations, our results suggest that the addition of PC results in decreased toxicity to the gastric mucosa in comparison to sulindac alone, and therefore supports the important role of PC in protecting gastric mucosa when associated with sulindac treatment. Further, the changes observed in blood counts (Table 2) suggest improvements in the anemia usually associated with polyp burden in the *Apc*^*min/+*^ model and is consistent with the polyp reduction observed in animals treated with sulindac or sulindac-PC.

Sulindac has been shown to inhibit β-catenin expression in the histologically normal appearing colon tissue of patients with the hereditary colorectal cancer syndromes, Hereditary Non-Polyposis Colorectal Cancer, also known as Lynch Syndrome, and FAP [35, 36]. Since the preparation of sulindac with phosphatidylcholine required sonication of the drugs, we confirmed biological activity of these preparations in vivo by demonstrating a reduction of polyp burden, significantly decreased nuclear β-catenin staining in the remaining polyps and a trend toward decreased urinary prostaglandins of treated mice. While PGEM, 2,3 dinor-TXB2 and 2,3 dinor-PGF1a appear to be decreased in sulindac and sulindac-PC treated mice, 11-dehydro-TXB2 only appears to be decreased in sulindac treated mice (Fig. 3c). One of the limitations of this analysis is our sample size. While we attempted to collect urine from each animal at the end of the study, we were unable to collect enough volume from many of the mice, resulting in pooled samples and overall reduced numbers. Specifically, we were only able to run prostaglandin levels on two samples for untreated mice, three samples each for PBS, PC and sulindac treated mice, and four samples for sulindac-PC treated mice. The low number of samples and variability of some measures preclude any formal statistical analyses of these data. However, the general decline in urinary prostaglandins of mice treated with either sulindac or sulindac-PC is supportive of systemic COX suppression. Our finding of significantly decreased nuclear β-catenin in remaining polyps is stronger evidence of the biological activity of sulindac and sulindac-PC and may suggest lower risk for these lesions to recur. Indeed, β-catenin, COX-2 and P53 staining have been used retrospectively, to show a significant association with adenoma recurrence in a prospective chemoprevention trial [37]. Together, these data support the efficacy, biologic activity and improved GI safety of sulindac combined with a phospholipid, lending critical support to the concept of improved safety for chemopreventive NSAIDs combined with phospholipids. If validated, these findings have the potential to significantly expand the portion of the population able to benefit from NSAID based CRC chemoprevention by reducing the risk of GI toxicity. Over time, such increasing use, combined with screening, may lead to profound reductions in CRC incidence.

In sulindac-PC, the sulindac is not covalently associated or crosslinked to the PC, rather the interaction is limited to ionic and hydrogen bonding and is expected to resemble the associations of aspirin-PC and indomethacin-PC as previously published [15]. Although not measured in our study, the association of sulindac and sulindac-PC is not expected to alter the bio-availability or pharmacokinetics/pharmacodynamics of sulindac, as has been demonstrated for aspirin-PC [13].

While our study supports chemopreventive efficacy and improved gastric toxicity of sulindac-PC, the very low incidence of gastric ulceration limits the strength of our conclusions on GI safety. Additionally, our study was conducted over a relatively short duration of 3 weeks. It is possible that treatment over a longer time period may have resulted in additional gastric injury in both control and experimental groups. Now that we have established a method to measure gastric injury in our mice, further studies are needed to determine the consequences of daily oral gavage of sulindac with and without PC for increasing time periods. The dose used in our study was equivalent to approximately 150 mg/day in an adult human [29], whereas a clinical trial in patients with FAP utilized 150 mg twice a day for primary prevention [34]. Increased dosing and extended treatment periods may have improved our ability to detect more substantial differences in gastric toxicity by treatment group.

## Conclusions

Although the *Apc*^*min/+*^ mouse model does not consistently recapitulate the chemopreventive effects of aspirin observed in humans [22–27], our results with sulindac-PC provide indirect evidence that the addition of PC improved the GI safety without compromising chemopreventive efficacy. Further, the recently reported aspirin-PC xenograft studies provide more direct evidence of the chemopreventive activity of aspirin-PC [15]. Taken together with prior in vitro, in vivo and clinical trial data, these studies support the consideration of phospholipid or some other polar/zwitterionic lipid, in combination with NSAID preparations to improve the GI safety profile without compromising efficacy.

## Data Availability

Data sharing is not applicable to this article as no datasets were generated or analysed during the current study.

## Abbreviations

ACN: Acetonitrile
ALT: Alanine transaminase
ANOVA: Analysis of variance
Apc: Adenomatous polyposis coli
ASA: Acetyl salicylic acid
AST: Aspartate aminotransferase
Alk Phos: Alkaline phosphatase
BUN: Blood urea nitrogen
COX: Cyclooxygenase
CRC: Colorectal cancer
CVD: Cardiovascular disease
FAP: Familial adenomatous polyposis
FDA: Food and drug administration
GI: Gastrointestinal
H&E: Hematoxylin and eosin
HSD: Honestly significant difference
IHC: Immunohistochemistry
min: Multiple intestinal neoplasia
n: Number of animals
NSAID: Non-steroidal anti-inflammatory drug
PBS: Phosphate buffered saline
PC: Phosphatidylcholine
PGEM: Prostaglandin E2 metabolite
PGF1a: Prostaglandin F1alpha
PGI2: Prostaglandin I2
PPI: Proton pump inhibitor
RBC: Red blood cells
SD: Standard deviation
TXB2: Thromboxane B2
WBC: White blood cells
